# Sleep, Performance, and Memory in Flies

**DOI:** 10.1007/s40675-014-0006-4

**Published:** 2015-02-05

**Authors:** Stephane Dissel, Krishna Melnattur, Paul J. Shaw

**Affiliations:** Department of Anatomy & Neurobiology, Washington University in St. Louis, 660 S. Euclid Ave, St. Louis, MO USA

**Keywords:** Sleep, *Drosophila*, Memory, Plasticity, Learning, Genetics

## Abstract

*Drosophila* has proven to be a powerful model to identify genes and circuits that impact sleep. While the majority of studies have primarily been interested in identifying manipulations that alter sleep time, a growing body of work has begun to focus on how changing sleep influences functional outcomes such as cognitive performance, structural plasticity, and metabolism to name a few. Evaluating sleep time provides an appropriate entry point into elucidating sleep function. However, it is not possible to fully understand how a manipulation has impacted sleep regulation without first establishing how it has affected the animals’ well-being. Synaptic plasticity and memory are important functional outcomes that can be used to asses an animal’s status. In this manuscript, we review recent advances in studies examining sleep, memory, and performance. We conclude that as *Drosophila* sleep researchers expand their analysis beyond sleep time, the opportunities to discover the function of sleep will be enhanced.

## Introduction

Sleep was first described in *Drosophila* by two independent groups ~14 years ago [1, 2]. Each of the inaugural papers began their analysis by demonstrating that quiescence episodes met the four historical criteria used to identify sleep (quiescence, increased arousal thresholds, rapid reversibility, and homeostasis) [3]. Interestingly, neither group relied exclusively upon behavioral criteria but extended their analysis to other important variables that are commonly studied in connection with sleep in mammals (e.g., ontogenetic changes, pharmacological perturbations, and molecular correlates). In the years since, ~100 papers have used genetic tools in the fly to identify genes and circuits that significantly modulate sleep time [4, 5]. However, a minority of these studies have extended their analysis to include other variables that have been linked with healthy sleep. Not surprisingly then, while ~40 genes have been found to alter some component of sleep time, little is known about precisely how these genetic manipulations impact sleep regulatory mechanisms.

With this in mind, it is worth noting that in the absence of an independent assessment of the animal’s ability to function properly, it is not possible to gain insight into whether the manipulation has enhanced or disrupted sleep regulatory processes. For example, a genetic manipulation could enhance the ability of sleep to carry out its function, thereby allowing the animal to sleep less [6]. Alternatively, the manipulation could disrupt the ability of the fly to obtain needed sleep [7]. Both types of disruption could result in a short-sleeping animal. Similarly, a mutation that reduces the ability of sleep to carry out its function may necessitate that a fly sleep longer to compensate for inefficient sleep [8]. However, a manipulation might increase sleep without enhancing the ability of sleep to carry out its function; or, better still, enhance sleep function [9, 10]. While increased sleep may or may not improve well-being, it should not result in obvious deficits. It seems inaccurate to classify a fly as long-sleeping if they are, in fact, sleeping poorly. Unfortunately, the field has not yet developed an alternative nomenclature that can distinguish between manipulations that enhance or disrupt sleep regulation independently from the amount of time the animal spends sleeping.

In the end, determining whether a manipulation has enhanced or disrupted sleep regulation requires that investigators extend their analysis beyond sleep. A number of independent variables can be considered when evaluating sleep mutants further including lifespan [11–14], neurotransmitters [15, 16], and metabolic markers [17–20] to name a few. However, given the overwhelming relationship between sleep and memory that has been found throughout the animal kingdom, we favor using learning and memory [7, 10, 21, 22] or synaptic markers [23–27] as the tools to independently assess the functional outcomes of a manipulation that alters sleep. In this manuscript, we will review the relationship between sleep, performance, and learning in flies.

## Evaluating Learning and Memory in *Drosophila*

There are few areas of *Drosophila* neurobiology that have been more successful than investigations into learning and memory. *Drosophila* memory assays can be generally broken down into those that use classical conditioning and those that use operant conditioning. In classical conditioning, two stimuli are repeatedly paired such that a previously neutral stimulus (bell) will, over time, induce a response (salivation) that was originally only induced by food (unconditioned stimulus). In operant conditioning, an animal engages in a behavior that is either rewarded or punished; whether the animal experiences the reward or punishment depends upon the animal’s own behavior. A brief description of the memory assays used in sleep research will be outlined below.

The success of the *Drosophila* memory field can be largely attributed to the use of aversive olfactory conditioning as the primary tool to identify genes, circuits, and microcircuits important for memory formation (for a complete review see [28]). Olfactory conditioning has been so successful, because it severely restricts the number of sensory stimuli that an animal is exposed to and must then process to form a memory [29]. In this paradigm, a group of 50 flies are exposed to an odor (odor-A) as the conditioned stimulus (CS+) and given a series of shocks (the unconditioned stimulus, US). The flies are then presented with a different odor that is not paired with a shock (odor-B; CS–). Finally, the flies are tested by allowing them to choose between the odor that had been paired with the shock (odor-A) and the CS– (odor-B). The flies are tested in the dark on a flat surface to ensure that their choice is not influenced by visual, phototaxis, or geotaxis cues [30]. Flies that have formed a memory will select odor-B and avoid the odor paired with the conditioned stimulus (CS +; odor-A). In a separate experiment, odor-B is paired with the electric shock (CS+) while odor-A serves as the counter-odor (CS–). This reciprocal training protocol ensures that non-associative odor preferences cannot be misinterpreted as associative learning. One experiment consists of eight replicates of 100 flies (50 w/ odor-A as the CS+ and 50 with odor-B as the CS+). Typically, 80 % of healthy flies will avoid the CS+, while only 20 % of memory-impaired flies will avoid the CS+ [31]. This assay has been reported to be highly sensitive to genetic background [32].

**Fig. 1:**
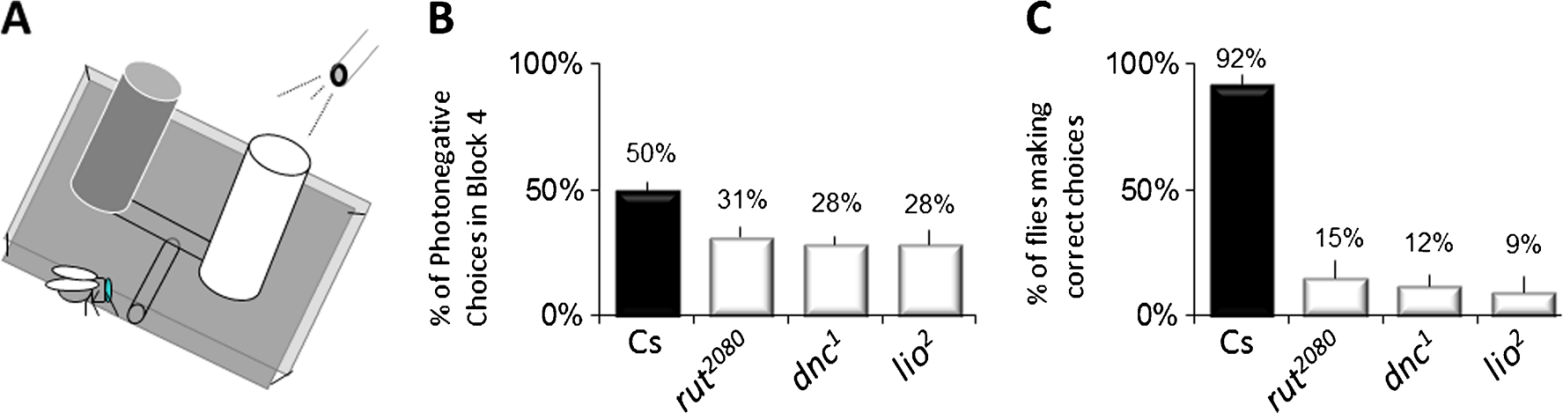
Aversive phototaxic suppression produces robust changes in performance. **a** Schematic of the apparatus. **b** Data for a single replicate of *n* = 8 flies for *Cs*, *rut* ^*2080*^, *dnc* ^*1*^, and *lio* ^*2*^ mutants. Data are expressed as the percentage of photonegative choices in block 4. **c** Data from 4 groups/8 flies expressed as a percentage of flies avoiding light/quinine

Perhaps because sleep may be less important for strongly encoded memories [33], or because sleep is believed to be important for integrating and processing complex information [34], the majority of *Drosophila* sleep studies have used multisensory operant conditioning assays. One such assay is an operant visual learning paradigm, the aversive phototaxic suppression (APS) [35]. In the APS, flies are individually placed in a T-maze and allowed to choose between a lightened and darkened chamber over 16 trials (Fig. 1a). Flies that do not display phototaxis during the first block of four trials are excluded from further analysis. During 16 trials, flies learn to avoid the lightened chamber that is paired with an aversive stimulus (quinine/humidity). The performance index is calculated as the percentage of times the fly chooses the dark vial during the last four trials of the 16 trial test. In the absence of quinine, where no learning is possible, it is common to observe flies choosing the dark vial once during the last four trials in block four. In contrast, flies never choose the dark vial two or more times during block four in the absence of quinine [36]. Thus, short-term memory (STM) is defined as two or more photonegative choices in block four [36].

In olfactory conditioning, flies are tested once and make a single choice. The performance index is calculated as the percentage of flies in a group that avoid the CS+. When tested using olfactory conditioning, ~80 % of healthy flies avoid the CS+ while only ~20 % of the classic memory mutants (*rutabaga*, *dunce*, and *linotte*) avoid the CS+ [31]. In the APS, each fly makes four choices in the last block. As a consequence, the behavior of each individual results in a quantitative score. An example of performance in *Cs*, *rutabaga*, *dunce,* and *linotte* can be seen in Fig. 1b. Note that the performance index seems much smaller in the APS (50 vs. 30 %) compared to olfactory conditioning (80 vs 20 %). However, the performance indices are calculated differently. If one conducts multiple replicates, as is done with olfactory conditioning, and expresses the performance index as the percent of flies that avoid the quinine/humidity, the performance index for the APS and olfactory conditioning look much the same (Fig. 1c). Indeed, power analysis yields a Cohen’s d of 1.8 [36]. Moreover, the performance of individuals is extremely stable over time [37]. Thus, while several hundred flies must be evaluated to generate a performance index using olfactory conditioning, only eight flies/genotype are needed to obtain statistical differences using the APS. Importantly, performance in the APS does not appear to be highly sensitive to genetic background [36].

The other most commonly used associative memory assay employed in *Drosophila* sleep research is courtship conditioning. In this assay, a male fly is exposed to an unreceptive female which will repeatedly reject the males’ courtship attempts. The rejection is aversive such that an operant association is formed between the courtship attempts and the rejection. Memory is demonstrated when a previously trained male spends less time courting during the test period than his naïve brothers. An important feature of this assay is that, it is clearly ethologically relevant. Moreover, the assay requires that flies process complex, naturalistic visual, tactile and pheromonal cues in conjunction with social behaviors and postures [38]. Perhaps because the assay depends upon naturalistic behavior, significant courtship scores can be obtained using 16–20 flies/group, and the resulting memory scores are robust and reproducible. Although baseline courtship levels can be influenced by genetic background [39], the ability to form a memory does not depend upon baseline courtship [40, 41]. Thus, like the APS, courtship conditioning is a multisensory operant assay that is not strongly confounded by genetic background. In contrast to olfactory conditioning, which requires the investigator to strictly limit all types of sensory stimuli and requires a dedicated room, courtship conditioning uses multisensory information, and therefore is not impacted by small variations in lighting (flies will learn both with and without light) or geotaxic cues, etc. Moreover, since the assay is not as sensitive to environmental cues, it does not require a dedicated room. Thus, any thoughtful *Drosophila* lab with a computer and a webcam can evaluate memory without the need to purchase expensive equipment and obtain additional space.

## Is Sleep Loss Bad?

When, almost 15 years ago, two independent laboratories showed that flies sleep, expectations that the function of sleep would be uncovered using *Drosophila* sleep-studies were raised. That is because *Drosophila* is an ideal model to study sleep and its relationship to other key physiological functions. Indeed, there are numerous well-established behavioral and molecular assays that can be used to assess cognitive performance, lifespan, metabolism, neuronal plasticity, etc. In addition, the power of *Drosophila* genetics ensures that genes involved in sleep regulation can be easily identified and manipulated in a circuit specific manner. Thus, it is somewhat surprising, that despite the discovery of ~40 genes that alter some component of sleep time, little is known about how sleep impacts the many physiological functions that are part of normal well-being. As mentioned above, we believe that in order to fully understand sleep and its function, the analysis of a given manipulation must be extended to at least one assessment that is independent of sleep. Given the well-known relationship between sleep and learning/memory in humans, we favor using cognitive performance as a way to further assess the consequences of genetic manipulations on sleep.

### Cognitive Performance in Short-Sleeping Flies

As previously stated, numerous genes are known to modulate sleep in *Drosophila* [4, 5]. In particular, many short-sleeping mutants have been described. Thus, studying the cognitive abilities of these short-sleeping mutants may provide a unique opportunity to elucidate sleep function. Surprisingly, few studies have evaluated memory in short-sleeping flies. One study that nicely evaluated performance, examined loss-of-function mutations in both the α (*Shaker*, *Sh*) and β (*Hyperkinetic*, *Hk*) subunits of a tetrameric voltage-dependent potassium channel [7]. When assessed for cognitive performance using an operant heat-box conditioning paradigm (in which flies have to learn and remember to avoid a spatial location associated with heat), short-sleeping *Sh* and *Hk* mutants showed intact learning but reduced short-term memory [7]. Thus, the short-sleeping phenotype of *Sh* and *Hk* mutants is associated with a negative cognitive outcome and suggests that neither mutant may be getting enough sleep. Another example comes from our recent work, in which we manipulated the expression level of antimicrobial peptides (AMPs) specifically in glia and in neurons [37]. We found that when we overexpressed *drosocin* (*dro*) in neurons, sleep was reduced, and this was associated with impaired STM. Interestingly, the expression of *Metchnikowin* (*Mtk*) in glia increased sleep. Thus, while we might have predicted that long-sleeping *Mtk-*expressing flies would have intact STM, we found that they were substantially impaired instead. Given that other long-sleeping flies (e.g., CREB and PKA mutants) are cognitively impaired [8], we expect that many long-sleeping flies will turn out to have inefficient sleep. Being able to distinguish between good and bad learning, long-sleeping flies will be important for understanding how sleep impacts the brain.

### Acute Sleep Deprivation may, or may not Induce Cognitive Deficits

Acute sleep deprivation can be performed on flies to assess sleep regulation as measured by sleep homeostasis. However, if a fly does not have a sleep rebound, it is not clear that they are better able to withstand wakefulness or if they can not initiate homeostatic mechanisms. Importantly, sleep deprivation disrupts STM as assessed by APS, courtship conditioning, and olfactory conditioning [21, 22, 42]. Thus, the assessment of STM can be used to gain additional insight into how the manipulation has impacted sleep regulation. For example, overexpressing the *Drosophila dopamine 1-like receptor* (*dDA1*) specifically in the mushroom bodies (MBs), an important learning and memory center in the fly brain allows the fly to maintain normal performance even after sleep deprivation [21]. Similarly, manipulating *Notch* signaling in the MBs also protects flies from sleep loss-induced memory impairments [43]. Interestingly, silencing the MBs during sleep deprivation prevents cognitive impairments as assessed using the olfactory conditioning [42]. Together, these data indicate that increased waking exerts its effects largely through the MBs.

Since sleep deprivation is known to be bad for the brain, much of the focus has been on genes that are primarily expressed in neurons such that genes involved in metabolism have not been well studied. However, flies mutant for *Lipid-storage Droplet-2*, a gene involved in lipid metabolism, are able to remain awake without suffering cognitive deficits. These data suggest that non-neural tissues may play important roles in regulating how an animal responds to sleep loss [17]. With this in mind, sleep deprivation and starvation differentially impacted STM in different mutant alleles of the *foraging* (*for*) gene which codes for protein kinase G (PKG) [41]. For example, flies with increased PKG *(rovers)*, are resistant to sleep loss as measured by both low sleep rebound and the ability to form STM after sleep deprivation. However, they became impaired following starvation. In contrast, flies with low PKG (*for* ^*s2*^), are impaired during baseline and following sleep deprivation but regained their ability to form STM following starvation [41]. This surprising observation would not have been possible without evaluating STM and emphasizes further the need to move beyond sleep time during characterization of a given mutant.

## Is Sleep Good?

Sleep deprivation studies have demonstrated that sleep loss impairs cognitive performance in flies and results in deficits in experience-dependent plasticity [21, 23, 24, 26, 38]. These data indicate that behavioral adaptation and structural plasticity are disrupted in the absence of sleep. However, by themselves, sleep deprivation studies cannot rule out the possibility that sleep simply plays a permissive role in facilitating changes in neural structure and function. Thus, in parallel with sleep deprivation studies, several laboratories have pursued alternative approaches to determine whether sleep plays an active and positive role in memory formation.

One alternative to sleep deprivation is to modulate plasticity directly; if plasticity and sleep are interdependent, then manipulations that increase plasticity should increase sleep. Although there are many ways to modulate plasticity, it has long been recognized that exposing animals to an enriched environment increases the number of synapses (reviewed in [38]). When flies were housed in socially enriched environments, they increased their sleep relative to their socially isolated siblings [22]. Another way to increase neuronal plasticity is to expose animals to training protocols that induce long-term memory (LTM). Not surprisingly, sleep was also dramatically increased when LTM was induced using courtship conditioning [22]. To determine whether the increase in sleep was due to nonspecific features of the training protocol (e.g., exercise) or to memory per se, flies were sleep-deprived for 4 h immediately following training. Even though the flies were awake for 4 additional hours, they did not exhibit either the typical post-training increase in sleep or a subsequent LTM. The ability to increase sleep by modulating the environment to induce plasticity has also been reported using different protocols [23]. Subsequent mechanistic investigations have used genetic tools to block plasticity [26, 27]. These experiments are complementary to post-training sleep deprivation; in that, they seek to test the hypothesis that the increase in sleep is due to non-specific environmental factors (i.e., independent upon plasticity). Interestingly, mutations in genes that are known to be important for neuronal plasticity prevent social enrichment and courtship conditioning protocols from either changing synapses or increasing sleep [25, 26]. Thus, plasticity is a strong modulator of sleep need.

The fact that plasticity can increase sleep is compelling. However, it is important to know whether sleep can, in turn, enhance plasticity. Recently, the dorsal fan-shaped body was identified as a major sleep-promoting center in the *Drosophila* brain [9]. Similar to the inaugural papers identifying sleep in flies, the characterization of sleep following fan shaped-body activation did not rely exclusively upon the historical criteria, but also evaluated other important variables that are commonly studied in connection with sleep in mammals. Results indicated that the sleep induced by fan-shaped body activation is homeostatically regulated, disrupted by caffeine, displays similar ontogenetic regulation, and modulates markers of synaptic plasticity similar to spontaneous sleep [9, 24, 44]. Despite these observations, there remained a remote possibility that the sleep induced by fan-shaped body activation is not truly sleep. Given the data presented above, plasticity was evaluated following genetically induced sleep. It has been known for some time that social enrichment disrupts the ability of flies to form LTM as assessed by courtship conditioning [45]. It is also known that sleep is required to downscale synapses that were increased following social enrichment [23, 25]. Thus, we hypothesized that if the sleep seen following fan-shaped body activation plays a role similar to spontaneous sleep, it should expedite the downscaling of synapses and restore the ability of the flies to form LTM. Indeed, sleep induction was shown to enhance the sleep-dependent decrease in synapses following social enrichment and restore LTM as predicted by the synaptic homeostasis hypothesis [9, 44].

Can sleep augment memories as has been predicted by human sleep studies [34, 46, 47]? To test this hypothesis, flies were trained using courtship conditioning. However, in contrast to the experiments described above, flies were trained using a protocol that is only capable of inducing STM. When sleep was induced thermogenetically by activating the fan-shaped body for 4 h immediately after training, STM was converted to LTM [9]. Thus, our data indicate that activating the fan-shaped body not only induces a state of sleep that meets all of the required historical criteria, it also modulates a number of other sleep-related variables appropriately and, importantly, it enhances memory consolidation. If we had not evaluated plasticity, we would never have known that sleep can convert STM into LTM. These data emphasize that sleep is sufficiently complex as to preclude using sleep time as the only and penultimate outcome variable to describe how a manipulation, of any kind, impacts sleep.

There are of course, caveats. Clearly, not every manipulation that increases sleep will be beneficial for performance. Indeed, one of the earliest reports on fly sleep showed that the mutations in the cyclic AMP pathway components—the *rutabaga* adenylyl cyclase and the cAMP response element binding protein CREB—increased sleep [8]. As mentioned above, recent work has shown that overexpression of the antimicrobial peptide *Metchnikowin* increases sleep and impairs STM formation as measured with the APS [37]. We do not believe that such a result will be uncommon. Thus, it is our view that the field would vastly benefit if more investigators would consider evaluating the functional outcomes of their manipulations. In this regard, it is interesting to note a recent report that demonstrated a sleep-promoting role for the neuropeptide sNPF [48]. Activating sNPF neurons dramatically increased sleep, yet appeared to block a homeostatic rebound to sleep deprivation. The authors suggested the intriguing hypothesis that sNPF neural activation induced local sleep. We believe that this is an exciting possibility that could be tested by evaluating learning and memory. If sleep induced by sNPF activation impairs memory or if it does not support memory following sleep loss, then the hypothesis might need further refinement. Surprisingly, we can find only one other manuscript that described a genetic manipulation that increases sleep and also evaluates memory [10]. In that report, the authors found that heat shock-induced overexpression of the *fatty acid binding protein* increased sleep and was also associated with improved performance in a 7-day olfactory memory task.

## Summary

**Fig. 2:**
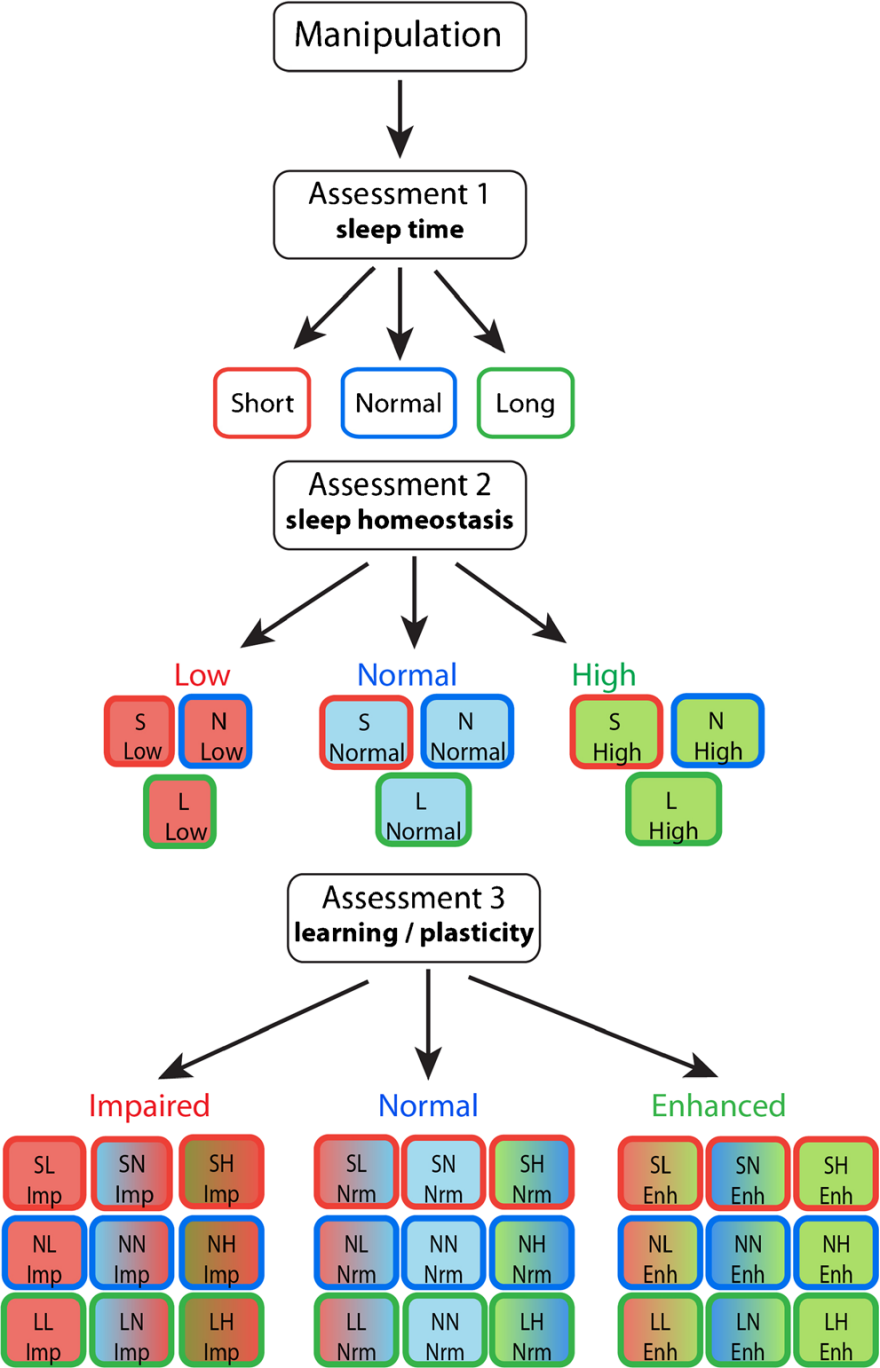
Flow chart to characterize sleep manipulations. We believe that sleep time is an insufficient metric to describe the effect of sleep manipulation on an animal’s physiology, and we therefore propose that a minimum of three assessments be made to characterize each manipulation. Baseline sleep time provides an easy starting point—flies are characterized as either short, normal, or long sleepers and marked with a *red*, *blue* and *green box* outline, respectively. The second assessment is sleep homeostasis—flies are characterized as having a low, normal, or high sleep rebound denoted with *filled boxes of colors red*, *blue, and green*, respectively. The level of sleep rebound is independent of the level of baseline sleep so each of the three categories of sleep rebound contains short (*S*), normal (*N*), or long (*L*) sleeping flies yielding a total of nine categories. A fly with low sleep rebound and normal baseline sleep, for example, is thus labeled “N Low” and marked with a *red box in a blue border*. We propose using memory as the third assessment—flies are characterized as having impaired normal or enhanced memory. This measurement is independent of the first two, yielding a total of 27 categories. As above, short, normal, and long levels of baseline sleep are denoted by *red*, *blue*, *and green box* outlines and denoted S, N, and L, respectively. Further, the box fill color for each category is color coded to reflect the outcomes of assessments 2 and 3. Thus, outcomes of low (*L*), normal (*N*), and high (*H*) sleep rebounds are marked with a *fill color on the left of red*, *blue*, *and green*, respectively, and outcomes of impaired (*Imp*), normal (*Nrm*), and enhanced (*Enh*) memory are marked with a *fill color on the right of red*, *blue*, and *green*, respectively. Within each memory outcome category, levels of baseline sleep are arranged in rows, and levels of sleep rebound in columns. A short-sleeping fly, with normal sleep rebound and impaired memory, for example, would be in row 1, column 2 of the “impaired” category, labeled “SN Imp”, and be marked with *box with blue on the left and red on the right with a red border*

*Drosophila* genetics has been successful at identifying genes and circuits that modulate sleep time [4]. Surprisingly, only a small number of *Drosophila* laboratories are specifically addressing questions pertaining to sleep function. Some of the work has focused on synaptic plasticity [9, 23–25, 27], while other studies have examined learning and memory [17, 21, 43] or metabolism [17, 19, 49, 50]. As mentioned above, it is not possible to fully understand how a manipulation has impacted sleep regulation without first establishing how it has affected the animals’ well-being. Certainly, synaptic plasticity, memory, and metabolism are important functional outcomes that can be used to asses an animal’s status. As mentioned, the field has not developed a nomenclature that can distinguish between manipulations that enhance or disrupt sleep regulation independently from the amount of time the animal spends sleeping. As a suggestion on how we, as a field, might proceed, we provide a flow chart in Fig. 2 that describes a systematic approach that can help place the outcome of genetic manipulations into the appropriate regulatory context. While sleep time is the most obvious starting point, it does not by itself, provide adequate information to assess sleep regulation. In order to assess sleep regulation, one must evaluate sleep homeostasis. Sleep homeostasis can be evaluated in any fly as long as baseline sleep is stable; the magnitude of the sleep rebound is not dependent upon total sleep time (e.g., short and long-sleeping flies exhibit similar sleep rebounds) [9, 14]. As seen in Fig. 2, after evaluating both sleep time and sleep homeostasis, there are nine possible outcomes. Unfortunately, sleep homeostasis, like sleep time, is ambiguous with respect to whether it indicates functional improvements or deficits. If one were to consider memory as one option to further clarify the animals’ status, there are 27 possible outcomes. Nine of these will have similar amounts of baseline sleep, nine will have similar sleep rebounds and nine will have similar learning outcomes. However, within a category, flies will all be different. Obviously, a single manipulation will not require that 27 categories be evaluated. Only three assessments must be made to place a mutant into a category that better describes its phenotype. Our previous data has shown that a mutant may be resilient to sleep loss while being vulnerable to starvation [41]. This does not reflect a limitation of the approach as much as it emphasizes that mutants must be better characterized in general. We do not wish to imply that the third assessment must be memory; it could be any variable that provides unique information about the animals’ well-being. Ultimately, we expect that as *Drosophila* sleep researchers expand their analysis beyond sleep time, in particular memory, the opportunities to discover the function of sleep will be enhanced.
